# Kinetic Modeling of Microbial Inactivation in Juices Treated With Pulsed Electric Fields (PEF): A Comparative Study on Microbial Heterogeneity and Food Matrix Effects

**DOI:** 10.1002/fsn3.72408

**Published:** 2026-10-07

**Authors:** Fotios Lytras, Georgios Psakis, Matthew Xuereb, Frederick Lia, Ruben Gatt, Javier Raso, Vasilis Valdramidis

**Affiliations:** ^1^ Faculty of Health Sciences, Department of Food Sciences & Nutrition University of Malta Msida Malta; ^2^ Malta College of Arts and Sciences, Institute of Applied Sciences, Main Campus Paola Malta; ^3^ Faculty of Science, Metamaterials Unit University of Malta Msida Malta; ^4^ Food Technology, Facultad de Veterinaria, Instituto Agroalimentario de Aragón‐IA2 Universidad de Zaragoza‐CITA Zaragoza Spain; ^5^ Department of Chemistry National and Kapodistrian University of Athens Athens Greece

**Keywords:** chemical analysis, juices, kinetic modeling, microbial inactivation, pulsed electric fields (PEF)

## Abstract

Pulsed electric field (PEF) processing is a promising technology for preserving fruit and vegetable juices. This research study evaluated the effect of pulsed electric fields (PEF) at 20 kV/cm on the microbial and quality parameters of pomegranate, tomato, and carrot juices. The inactivation kinetics of *Escherichia coli* strains (K12 and FAM 21843) and *Saccharomyces cerevisiae* strains (AD1890 and 130.0014) were fitted using a global model, which combined a log‐linear primary model with a second‐order polynomial. for describing the polyphasic inactivation kinetics. Maximum microbial inactivation rates (*k*_*max*_) increased with the increase of *Wτ*, reaching > 5‐log reductions for *E. coli* strains in pomegranate and carrot juice, and > 3.85‐log reduction in tomato juice. Among strains, *S. cerevisiae* 130.0014 showed the highest *k*_*max*_ values above > 80 kJ/kg in pomegranate and tomato juices. Minor differences were observed at high *Wτ* levels among *E. coli* strains in pomegranate juice and among *S. cerevisiae* strains across all the food matrices. Furthermore, the occurrence of sublethal injury was low to *E. coli* strains under PEF, independent of the food matrix, within pomegranate juice, showing the highest levels. Quality analysis of all juices revealed that PEF did not affect antioxidant compounds or their antioxidant activities, as no statistically significant values (*p* < 0.05) were identified. Overall, the effectiveness of PEF in microbial inactivation is primarily governed by *Wτ*, with some food‐matrix‐dependent variations. These findings highlight PEF's potential as a food processing alternative that preserves quality while achieving microbial inactivation.

## Graphical abstract

Kinetic Modelling of *Escherichia coli* and *Saccharomyces cerevisiae* strains in fruit and vegetable juices.

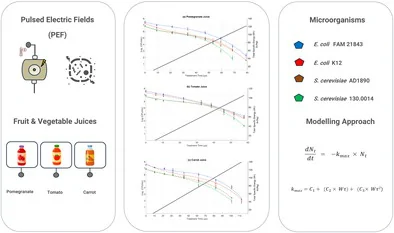

## 1 Introduction

With improvements in living standards, growing awareness of nutrition and health, and the acceleration of lifestyle pace, there is increasing demand for natural foods with high nutritional value to meet evolving consumer dietary preferences (Niu et al. 2024). Fruit and vegetable juices have gained popularity as low‐calorie, health‐promoting beverages rich in bioactive compounds (Lan et al. 2023; Martins et al. 2020). These juices are rich in essential micronutrients (vitamins and minerals, dietary micronutrients, sugars, acids) and bioactive compounds (polyphenols, flavonoids, carotenoids, and various other organic components) (Lan et al. 2023).

During processing, juices can become contaminated by different microorganisms from various sources (raw materials, washing waters, etc.) and potentially can cause foodborne diseases (Lee et al. 2021). To ensure safety but also quality, the juice and beverage industry typically employs conventional thermal treatments for the inactivation of pathogenic and spoilage microorganisms as well as endogenous enzymes. Although effective, intense heat treatments can facilitate the degradation of bioactive compounds and vitamins (Brito and Silva 2024). In response, “non‐thermal technologies” such as PEF have emerged as alternative treatments to thermal processing due to the achievement of microbial inactivation of vegetative cells of bacteria and yeasts, in parallel with ensuring the preservation of sensory and nutritional qualities of fresh products (Delso et al. 2022).

The microbial inactivation due to PEF is based on the electroporation phenomenon and its ability to provide adequate cellular stress, which leads to inactivation of target microorganisms (Kotnik et al. 2012; Mahnič‐Kalamiza and Miklavčič 2022). Microbial cells that are typically exposed to an electric field strength above 10 kV/cm and treatment times from micro‐ to milli‐seconds, resulting in membrane disruption and potentially subsequent microbial inactivation (Heinz et al. 2001; Roobab et al. 2018; Toepfl 2012). However, the efficiency of microbial inactivation is based on different factors such as: the microbial characteristics including the type of microorganism, the species, the strain and culture conditions (Álvarez et al. 2002; Heinz et al. 2001; Lytras et al. 2024; Saldaña et al. 2009), as well as food product characteristics such as pH and conductivity (García et al. 2005; Saldaña et al. 2009) and processing parameters including electric field strength, total specific energy (Saldaña et al. 2010b; Thamsuaidee et al. 2024).

Increasing evidence indicates that microbial responses to PEF are heterogeneous, with substantial variability in resistance observed not only between species but also among strains within the same species (Lytras et al. 2024). Thus, this strain‐dependent heterogeneity complicates accurate prediction of microbial inactivation and the development of robust, broadly applicable kinetic models. In addition, variations in the physicochemical properties of food matrices modulate microbial responses to PEF, implying that microbial inactivation kinetics derived from one product potentially cannot be directly extrapolated to other food systems.

In addition, electroporation caused by PEF treatment does not directly correlate with cell death, as reversible pores can be formed, allowing microbial membrane re‐sealing and survival. These sub‐lethally injured cells represent a food safety concern, as they can potentially recover during storage and compromise product stability and safety (Garcia et al. 2006; Saldaña et al. 2010a).

Mathematical modeling can help in predicting microbial inactivation kinetics under PEF treatment, thereby facilitating the design of stable and safe products while reducing the need for extensive processing trials (Álvarez et al. 2003; Wouters et al. 2001). These mathematical models associate different PEF treatment parameters with the proportion of surviving microorganisms to predict microbial inactivation levels (Singh et al. 2017). Survival curves typically exhibit a first‐order log‐linear pattern for small log reductions. Although primary model parameters are typically estimated separately for each condition, and then linked to process variables through secondary models. In contrast, in this study both primary and secondary models are fitted simultaneously to the full dataset. This allows the assessment of the effects of processing conditions and juice matrix characteristics on microbial inactivation to be captured directly within one modeling step. This approach offers several practical advantages, including reduced propagation of errors from stepwise fitting, better use of the available data, and a more direct assessment of how process variables, microbial strains, and juice types influence inactivation behavior and their potential interactions. However, when the curves deviate from this pattern, alternative mathematical prediction models are necessary (Rivas et al. 2006). To date, various kinetic models have been employed to describe different microorganisms' inactivation by PEF in liquid foods including: first‐order kinetic models (Amiali et al. 2007; M. Rodrigo, Rodrigo, and Ruı 2003), the Bigelow model (Rivas et al. 2006; Singh et al. 2017), the Hulsgeger's model (Rivas et al. 2006; Singh et al. 2017; Zhong et al. 2005), the Fermi's model (Zhong et al. 2005), the Arrhenius model (Alkhafaji and Farid 2008), the Weibull distribution model (Rivas et al. 2006; Singh et al. 2017; Timmermans et al. 2014), the Peleg model (Singh et al. 2017), the Log–logistic models (Raso et al. 2000), and the quadratic response model (Mosqueda‐Melgar et al. 2007; Saldaña et al. 2011).

The objective of this work was to implement a comprehensive analysis of microbial inactivation kinetics using a global fitting approach, enabling the simultaneous modeling across multiple datasets and treatment conditions. Therefore, this research focused on characterizing and comparing the kinetic inactivation patterns of *E. coli* K12, *E. coli* FAM 21843, *S. cerevisiae* AD1890, and *S. cerevisiae* 130.0014 subjected to PEF treatments, with emphasis on strain‐dependent resistance, sub‐lethality (for *E. coli* as a food safety indicator), and food matrix effects. By examining inactivation kinetics under different food matrices, the study aims to assess the variability in microbial resistance to PEF. While secondary, the research assessed the effects of PEF on the chemical properties of these juices, including phenolic and flavonoid compounds, to balance microbiological and chemical analysis for food preservation. Ultimately, this work could contribute to the optimization of PEF processes, either alone or in use with other preservation methods, for industrial applications.

## 2 Materials and Methods

### 2.1 Strains, Medium and Culture Conditions

In this study, two wild types (WT) of two microbial species, one pathogenic and one spoilage, were used, namely *E. coli* K12 (BW25113), *E. coli* FAM 21843, *S. cerevisiae* AD1890 and *S. cerevisiae* 130.0014 (derived from different origins: *E. coli* K12 from NBRP *E. coli* Japan, *E. coli* FAM 21843 from raw milk cheese, *S. cerevisiae* AD1890 from fruit and *S. cerevisiae* 130.0014 from the food industry as previously mentioned by Battaggia et al. (2024); Lytras et al. (2024); Okafor et al. (2024); Tsagkaropoulou and Karatzas (2024)). All *E. coli* and *S. cerevisiae* strains were stored at −80°C in glycerol (30:70 v/v), (Merck, Germany). The *E. coli* and *S. cerevisiae* strains were reactivated and cultured with the same media and culture conditions as previously described Lytras et al. (2024). Following incubation to early stationary phase, the cultures of *E. coli* and *S. cerevisiae* strains were centrifuged (3000 x *g*) for 20 min and washed with Phosphate Buffer Saline solution (PBS, Oxoid, United Kingdom). Hereafter, the pellets of each strain were resuspended in pomegranate, tomato, and carrot juices accordingly before being exposed to PEF.

### 2.2 Pomegranate, Tomato and Carrot Juices

For this study, fresh produce was obtained from Mgarr Farming Ltd. (Mosta, Malta), in which pomegranate juice composed of a blend of three varieties (*Punica granatum var. Wonderful, Bullar, and Helu*), tomato juice (*Solanum lycopersicum var. Feiza*), and carrot juice (*Daucus carota subsp. sativus var. Nantes*). All juices were supplied with suspended solids removed during industrial processing under refrigerated conditions and stored at −20°C on arrival. Carrot juice was diluted with sterile deionized water at a ratio of 30:70 (v/v) to adjust the total soluble solids concentration and electrical conductivity. A Brix° refractometer (Atago, Japan) was used for the measurement of the total soluble solid content. The pH and electrical conductivity were measured with a benchtop multi‐parameter‐meter (Hanna Instruments, model H5522, Romania) (Table 1). The instruments were calibrated prior to measurements following the manufacturer's standard calibration procedures.

**TABLE 1:** Physicochemical properties of juices.

Fruit and vegetable juices	Density (*ρ*) (kg/m^3^)	Conductivity (ms/cm) (at 20°C–25°C)	pH (at 20°C–25°C)	Brix° (at 20°C–25°C)
Pomegranate juice	1060	3.9 ± 0.1	3.1 ± 0.1	12.0°
Tomato juice	1015	4.8 ± 0.1	4.4 ± 0.1	5.1°
Carrot juice	1010	3.1 ± 0.1	6.1 ± 0.1	3.0°

### 2.3 Continuous Flow PEF Processing System

In this study, the EPULSUS‐BM1A‐12 (3 kW, Energy Pulse System Lisbon, Portugal) pulse generator was used. This system has the capability of providing monopolar square wave pulses (1–200 μs), with a maximum output voltage of 12 kV, and a current of 200 A and frequencies of up to 200 Hz. For this study, a continuous mode (at one step without recirculation) at a flow rate of 6 L/h was used, pumping the sample through a treatment chamber equipped with parallel titanium electrodes separated by a 0.4 cm gap (3 cm length and 0.5 cm width), as described Lytras et al. (2024). This configuration resulted in a residence time of 0.36–0.38 s. Before PEF treatment, samples were tempered to an inlet temperature of 20°C using a coil immersed in a water bath circulation system (Witeg, Germany). After PEF treatment, the samples were cooled to ambient temperature using a cooling coil. The sample spent < 1 s in the pipeline connecting the treatment chamber's exit to the cooling heat exchanger, and 2.5 s. within the cooling heat exchanger. After collection, the sample was collected (sterile Falcon tube) and immediately immersed in an ice bath until the temperature dropped (below 20°C) (Figure 1). The actual voltage applied during processing was monitored through an integrated oscilloscope from Pico Technologies.

**FIGURE 1:**
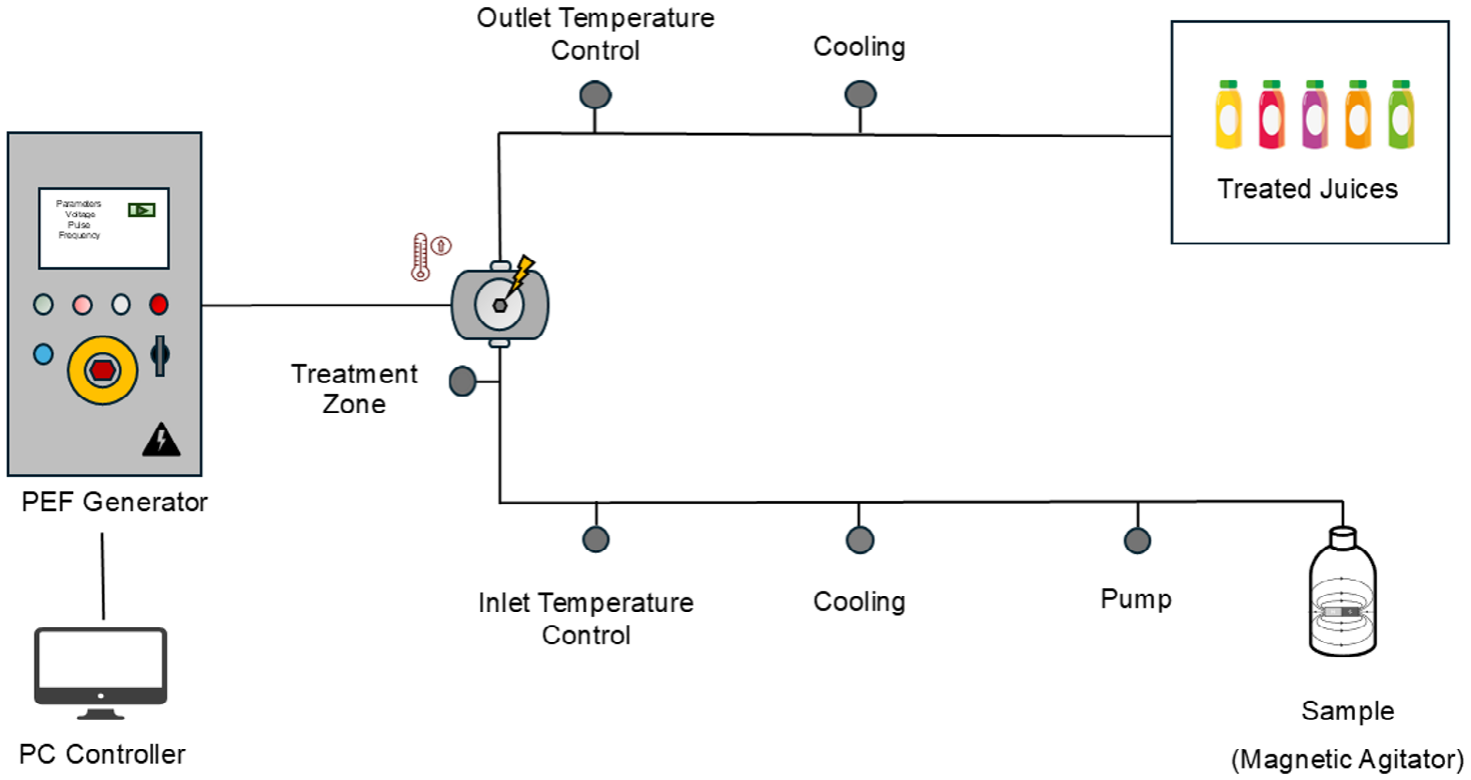
Set‐up of PEF equipment for a continuous process for microbial inactivation of juices.

Inlet and outlet temperature were monitored with a thermocouple (OM‐HL‐EH‐TC, OMEGA, Netherlands). Square pulses (4 μs width) were applied at an electric field strength of 20 kV/cm, with repetition rates from 4 to 78 Hz, resulting in outlet temperatures between 20 to 55.8°C. The duration of the treatments were calculated from theoretical values (residence time × frequency × pulse width) leading to treatment time between 0 and 112.3 μs, respectively. The total specific energy (*Wτ*) for the three products tested was between 0 to 139.2 kJ/kg influenced by the PEF treatment and juice conductivity (Table 2). The estimated values were calculated starting with the calculation ofthe specific energy input per pulse (*W*) using Equation (1) and followed by application of Equation (3) (as previously described Raso et al. 2016).

(1) $$W=\frac{1}{m}\int_{0}^{\infty }\frac{U\;{\left(t\right)}^{2}}{R}\mathrm{dt}=\frac{1}{m}\int_{0}^{\infty }U\left(t\right)\;I\left(t\right)\mathrm{dt}$$

where *m* is the mass (treated sample), *U*(*t*) and *I*(*t*) are the voltage across and current through the treatment chamber load at time *t*. The electrical resistance of the treatment chamber is described by *R* (in Ω), which can be calculated accordingly:

(2) $$R=\frac{1}{\sigma }\frac{L}{A}$$

where *σ* described the electrical conductivity of the treated food product (S/m) and *A* is the electrode area (m^2^).

**TABLE 2:** PEF treatments of pomegranate, tomato, and carrot juices.

Sample	Flow rate (L/h)	Electric field strength (kV/cm)	Total specific energy (kJ/kg)	Pulse width (μs)	Frequency (Hz)	Treatment time (μs)	Pulse number (n)	Outlet temperature (°C)
Pomegranate juice								
1	6	0	0	0	0	0	0	20
2	6	20	14	4	6	9.12	2.28	23.9
3	6	20	32.66	4	14	21.28	5.32	29.2
4	6	20	51.32	4	22	33.44	8.36	24.4
5	6	20	60.65	4	26	39.52	9.88	37
6	6	20	72.32	4	31	47.12	11.78	40.3
7	6	20	81.65	4	35	53.2	13.3	42.9
8	6	20	90.98	4	39	59.28	14.82	45.6
9	6	20	104.98	4	45	68.4	17.1	49.5
10	6	20	121.31	4	52	79.04	19.76	54.1
Tomato juice								
1	6	0	0	4	0	0	0	20
2	6	20	10.77	4	4	5.92	1.48	22.7
3	6	20	18.85	4	7	10.36	2.59	24.7
4	6	20	40.39	4	15	22.2	5.55	30
5	6	20	59.24	4	22	32.56	8.14	34.7
6	6	20	70.01	4	26	38.48	9.62	37.4
7	6	20	83.48	4	31	45.88	11.47	40.7
8	6	20	94.25	4	35	51.8	12.95	43.4
9	6	20	105.22	4	39	57.72	14.43	46.1
Carrot Juice								
1	6	0	0	0	0.0	0	0	20
2	6	20	12.5	4	7	10.08	2.52	23.1
3	6	20	26.78	4	15	21.6	5.4	26.9
4	6	20	39.28	4	22	31.68	7.92	30.1
5	6	20	55.35	4	31	44.64	11.16	34.2
6	6	20	76.78	4	43	61.92	15.48	39.7
7	6	20	101.78	4	57	82.08	20.52	46.2
8	6	20	124.99	4	70	100.8	22.68	52.1
9	6	20	139.28	4	78	112.32	28.08	55.8

The total specific energy input (*W*_*T*_, kJ/kg) can be calculated by multiplying *W* by the number of pulses applied:

(3) WΤ=W×n

### 2.4 Enumeration of Viable Cells

The untreated and treated cell suspensions were diluted in PBS. The diluted sample (0.1 mL or 1 mL divided at three different plates) was spread into the Petri dish. For each experimental condition three biological replicates were performed each originating from separate culture preparations, and for each biological replicate, two technical replicates (plating repeats) were conducted. For the enumeration of viable *E. coli* cells, two different media were used, incubated for up to 48 ± 2 h at 37°C: a nonselective medium Tryptic Soya Agar (TSA) and a selective medium Tryptone Bile X‐Glucuronide Agar (TBX) to identify the effect of PEF treatments and potential sub‐lethal population as described by Verhaegen et al. (2015). While for the enumeration of viable *S. cerevisiae* the medium of Malt Extract Agar (MEA) incubated for 48 ± 2 h at 30°C was used. Colony counts corresponding to the viable microorganisms were expressed as colony forming units per milliliter (CFU/mL).

### 2.5 Kinetic Modeling Analysis

Following the microbial survival studies, the kinetics of *E. coli* and *S. cerevisiae* strains were studied under varying total specific energies (*Wτ*) of PEF treatments applied to three different food matrices: pomegranate, tomato, and carrot juices. Microbial kinetic parameters were estimated using a global modeling approach as described in previous work by Lytras et al. (2026), that simultaneously fits primary and secondary models to the experimental data. The primary model employed a log‐linear model (Equation 4) (Geeraerd et al. 2005), while the secondary model employed was a polynomial type model (Equation 5) and was used to determine the total specific energy dependence of *k*_*max*_.

(4) $$\frac{dN_{t}}{\mathrm{dt}}=-k_{\max}\times N_{t}$$

*N*(*t*) (CFU/mL) is the microbial population at time *t*, and *k*_*max*_ (1/min) is the specific microbial inactivation rate.

(5) $$k_{\max}=C_{1}+\left(C_{2}\times W_{Τ}\right)+\left(C_{3}\times W_{T}^{2}\right)$$

$W_{Τ}:$ is the total specific energy, *C*_1_: is the baseline inactivation constant, *C*_2_: is the linear sensitivity coefficient, *C*_3_: captures the quadratic coefficient (nonlinear) effects.

Regression analyses were conducted to estimate the primary and secondary model parameters using Matlab Version 6.1 (The Mathworks, MA, USA). Furthermore, parameter optimisation was carried out with the use of the function Isqnonlin from the Matlab Optimisation Toolbox. The model's performance was assessed using metrics including Mean Square Error (*MSE*) and Regression coefficient (*R* ^2^) (Equations 6 and 7). The regression coefficient (*R* ^2^) value is from 0 to 1 (0: indicates a poor fit and 1: represents a perfect fit). This reflects the proportion of the variance in the data as described by the model of Den Besten et al. (2006). The regression coefficient *R* ^2^ is defined as:

(6) $$R^{2}=1-\frac{\mathrm{RSS}}{\mathrm{TSS}}$$

where RSS is the residual sum of squares and TSS is the total sum of squares.

The *MSE* is related to the differences of the experimental data and the estimated values of a model (Buzrul and Alpas 2004). Therefore, the higher the *MSE* value, the higher the discrepancy between the experimental data and the predicted output of the model. As a result, a lower value of the *MSE* predicts a better fitting capacity and a better model performance (Equation 7). *MSE* is defined as follows:

(7) $$\mathrm{MSE}=\frac{1}{n}\sum_{i-1}^{n}{\left(y_{\exp}\left(t_{i}\right)-y\left(t_{i},p_{\mathrm{ls}}\right)\right)}^{2}$$

where n is the total number of observations, $y_{\exp}\left(t_{i}\right)$ is the experimental data and $y\left(t_{i},p_{\mathrm{ls}}\right)$ is the predicted value from the related mathematical model.

The Akaike information criterion (*AIC*) is related to the evaluation of how well the model fits the experimental data. The smaller the *AIC*, the better the corresponding model's ability to describe the data (Johnson Esua et al. 2022).

(8) $$\mathrm{AIC}=2k+n\left[\ln\left(2\pi \cdot \mathrm{MSE}\right)+1\right]$$

where *n* is the total number of observations made, *k* is related to the number of model parameters, *MSE* (Mean Squared Error) and *SSE* (Sum of Squared Errors).

### 2.6 Chemical Analyses (Determination of Total Phenolic Content (TPC), Total Flavonoid Content (TFC), Total Ortho‐Diphenoland Cupric (TdOPC) and Cupric Reducing Antioxidant Power Assay (CUPRAC))

The total phenolic content (TPC), Total Flavonoid Content (TFC), Total Ortho‐Diphenolic Content (TdOPC) and Cupric Reducing Antioxidant Power Assay (CUPRAC) in the carrot, tomato and pomegranate juices before and after PEF treatments were measured as previously described Xuereb et al. (2025). Three independently processed samples were analyzed for each condition. The total phenolic content (TPC) was measured using the Folin–Ciocalteu (F–C) colorimetric method. In the case of pomegranate juice, a 10‐fold dilution was applied in order to reduce the measured absorbance below a value of 2. For the total flavonoid content, a quantity of 20 μL of the juice was combined with 10 μL of 10% aluminum chloride, 10 μL of 7% w/v sodium nitrite, and 80 μL of distilled water. The microtiter plate was then left at room temperature for 30 min, followed by the addition of 100 μL of 1 M NaOH solution. The calibration curves and graphs can be found in the Supporting Information (Figures S4–S7).

### 2.7 Statistical Analysis

Three biological replicates were used for each strain and condition in the microbiological experiments. For the chemical analyses, three independently processed samples were analyzed for each condition. For the parameter estimated data (*C*_1_, *C*_2_, *C*_3_), one‐way Analysis of Variance (ANOVA) and Fisher's LSD post hoc test were performed using GraphPad Prism 10.0.2 (GraphPad Software, San Diego, California, United States) for the identification of significant differences between *C*_1_, *C*_2_, *C*_3_. Multiplicity adjusted *p value* was calculated for each comparison as described by (Westfall et al. 2000; S.Paul Wright 1992). The differences between the parameter's estimated values lower than *p* < 0.05 were considered significant. Furthermore, for the chemical analysis, the approach followed the identification of statistical differences (*p* < 0.05) between untreated and treated samples was one‐way Analysis of Variance (ANOVA) and Dunnett's post hoc test.

## 3 Results and Discussion

### 3.1 Microbial Inactivation Kinetics and PEF Parameters

This study explored the microbial inactivation of four strains including *E. coli* K12, *E. coli* FAM 21843, *S. cerevisiae* AD1890, and *S. cerevisiae* 130.0014 when subjected to PEF treatments at 20 kV/cm with exposure times ranging from 0 to 112.3 μs, total specific energies from 0 up to 139.2 kJ/kg, and outlet temperatures between 20°C and 55.8°C, using pomegranate, tomato, and carrot juices as food matrices. This intensity was selected to align with typical industrial PEF operations, which generally apply electric field strengths from 10 to 20 kV/cm to ensure effective microbial inactivation (Toepfl 2012). Additionally, to closely mimic the industrial conditions, a continuous processing system was intentionally employed, similar to our previous study (Lytras et al. 2024).

As illustrated (Figure 2), microbial inactivation for both *E. coli* and *S. cerevisiae* strains intensified progressively with increasing total specific energy (as well as treatment time), highlighting the dynamic relationship between PEF parameters and microbial resistance across diverse juice environments. To date, several studies have demonstrated the association between microbial inactivation with increased: treatment time (Thamsuaidee et al. 2024) and total specific energy (Lytras et al. 2024; Timmermans et al. 2014). Moreover, PEF microbial inactivation studies have shown efficiency for the processing of various juices and the inactivation of microorganisms (Gurtler et al. 2010; Saldaña et al. 2011; Singh et al. 2017). In our study, the highest PEF conditions (total specific energies) were applied for each juice to assess the microbial inactivation for both strains of *E. coli* (K12 and FAM 21843) which serve as potential target microorganisms, showed a log reduction > 5 under PEF treatment (20 kV/cm, 79.04 μs, 121.31 kJ/kg, 54.1°C) for pomegranate juice, a log reduction > 3.85 under PEF treatment (20 kV/cm, 57.72 μs, 105.22 kJ/kg, 46.1°C) for tomato juice and > 5 a log reduction under PEF treatment (20 kV/cm, 112.32 μs, 139.28 kJ/kg, 55.8°C) for carrot juice, underlying high microbial inactivation efficiencies across all the juices. Similarly, a study by Evrendilek (2017) has shown that a 5.43 log reduction was achieved in pomegranate juice that was PEF‐treated (30 kV/cm, 65.3 J, 40°C), unraveling the potential of PEF processing for the microbial decontamination of pomegranate juice over conventional pasteurization treatments. The level of microbial inactivation that meets the minimum industry standard to approve PEF for commercial juice pasteurization, as per the Food and Drug Administration (FDA), is a 5‐log reduction for most resistant foodborne pathogens (U.S. Food and Drug Administation 2000).

**FIGURE 2:**
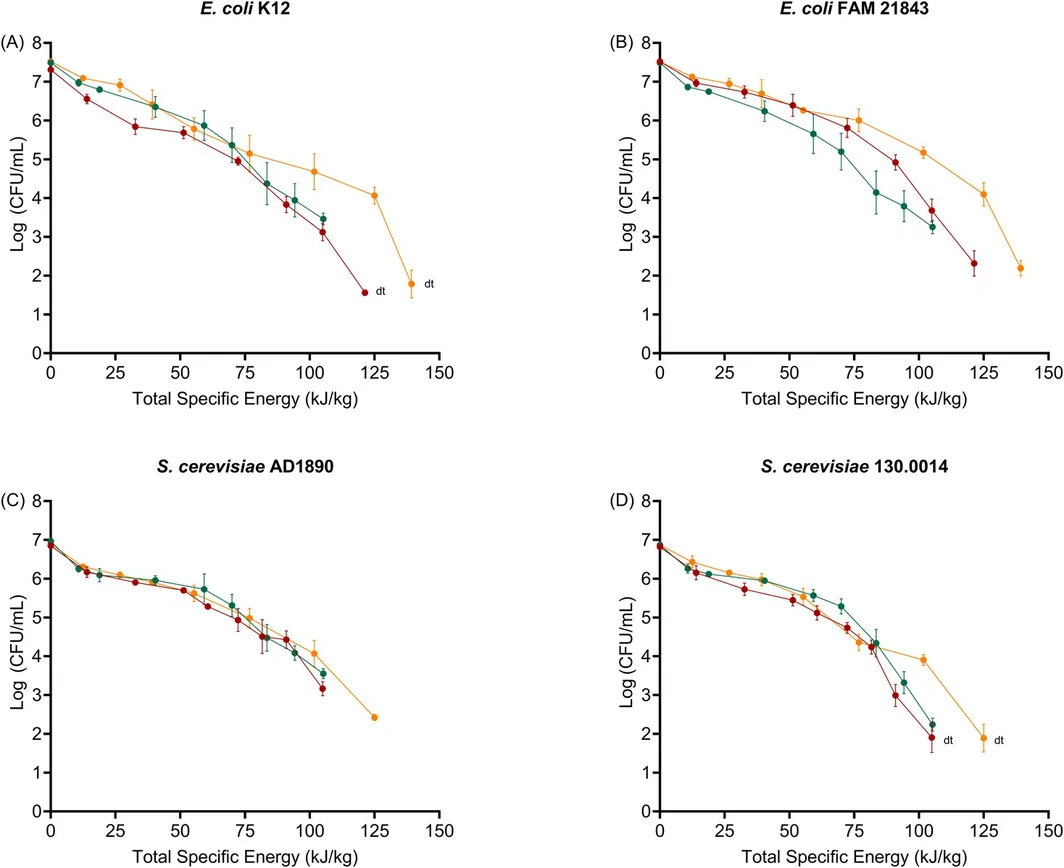
Survival curves of *E. coli* K12 (Α), *E. coli* FAM 21843 (Β), *S. cerevisiae* AD1890 (C), and *S. cerevisiae* 130.0014 (D) based on experimental data describing the changes in Log CFU/mL with total specific energy (kJ/kg) in pomegranate (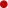), tomato (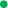), and carrot (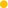) juices. Points with dt indicate exceedance of detection limit. Error bars indicate standard deviations on the log‐transformed data.

### 3.2 Sublethal Injury Effects

PEF treatments, similar to most of the environmental stresses, do not lead to an “all or nothing effect” to a microbial population (Wang et al. 2022). The sublethal injury is an important consideration in PEF‐treated foods because injured cells may recover during storage, posing safety risks (Jaeger et al. 2009). The differences in colony‐forming unit counts between selective (TBX) and nonselective (TSA) media for *E. coli* K12 and FAM 21843 in pomegranate, tomato and carrot juices are presented at Figure 3. In this study, the largest differences in colony‐forming unit counts between selective (TBX) and nonselective (TSA) media for *E. coli* K12 and FAM 21843 were observed only in pomegranate juice during medium‐energy PEF treatments (47.12–68.4 μs and 72.32–104.98 kJ/kg), indicating a higher proportion of sub‐lethally injured cells under these conditions. For example, under PEF treatment of 47.12 μs, 72.32 kJ/kg *E. coli* K12 showed log reduction of 2.36 (TSA) and 3.13 (TBX). In contrast, in tomato and carrot juices, *E. coli* showed minimal differences, suggesting fewer injured cells or more complete inactivation. A study by Zhao et al. (2011) showed that reversible permeabilization of cells occurred at approximately 20% and 10% when treated in phosphate buffer (10 mM, pH 7.0) with pulsed electric fields (PEF) at 15 kV/cm and 20 kV/cm, respectively, while PEF intensities above 25 kV/cm caused irreversible permeabilization, leading to permanent membrane damage. Additionally, due to their antimicrobial properties, juices can affect microorganisms and may have a potential synergistic effect when combined with pulsed electric fields (PEF). For example, a study by Ibrahim et al. (2011) demonstrated the antimicrobial activity of pomegranate juice against the growth of *E. coli* , while another study showed that tomato juice exhibits potent antimicrobial activity against *Salmonella typhi* (Kwon et al. 2024). Furthermore, detailed studies revealed that sub lethally membrane‐injured population of *E. coli* (NCTC 5934) after PEF treatment is heterogeneous, with less than 5% repair their membranes immediately, while the remaining 95% require energy and lipid synthesis for repair (Garcia et al. 2006). The recovery of the sub lethally damaged cells and their potential survival depended on the temperature, the pH of the medium and/or the incubation time after PEF treatment (Raso et al. 2016), indicating that the recovery of sub‐lethally injured cells is dependent on various parameters.

**FIGURE 3:**
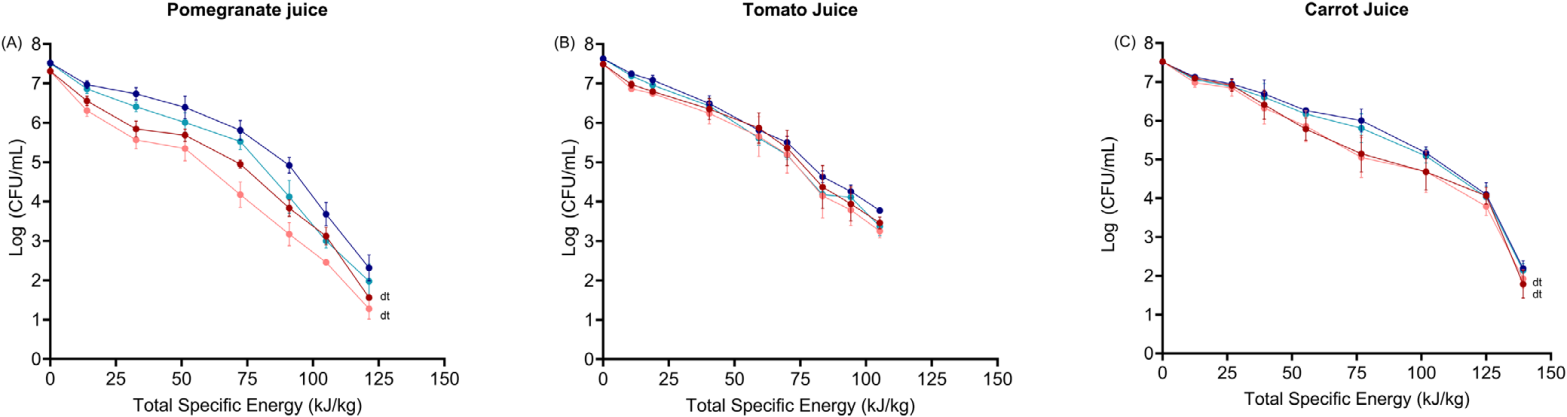
Survival curves of *E. coli* K12 plated on TSA (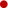) and TBX (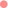) and *E. coli* FAM 21843 plated on TSA (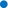) and TBX (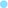) based on experimental data describing the changes in Log CFU/mL with total specific energy (kJ/kg) at pomegranate (A), tomato (B), and carrot (C) juices. Experimental data and error bars represent the mean values of three biological replicates and standard deviations on the log‐transformed data.

### 3.3 Modeling of Microbial Inactivation Kinetics

In this study, the survival profiles of the tested strains revealed a complex, nonlinear, multi‐phase inactivation pattern across various PEF treatments and food matrices (Figure 4). Earlier studies under PEF treatment observed a linear relationship in survival curves (Heinz et al. 1999; Reina et al. 1998). However, this may have been related to the limited inactivation levels < 4.0 log, which occurred only when the PEF treatments were applied at low electric field strengths or for short durations. Similarly, with our study, more recent research on PEF treatments of juices and various microorganisms (including the species of *E. coli* and *S. cerevisiae* ) has shown a nonlinear behavior (D. Rodrigo, Barbosa‐Cánovas, et al. 2003; Singh et al. 2017; Timmermans et al. 2014). To more accurately describe this nonlinear behavior, we applied a global kinetic modeling approach that explicitly accounts for the polyphasic nature of the inactivation profiles. In this approach, a log‐linear primary model was combined with a second‐order polynomial function, with treatment time, total specific energy and survival data incorporated as incorporated variables.

**FIGURE 4:**
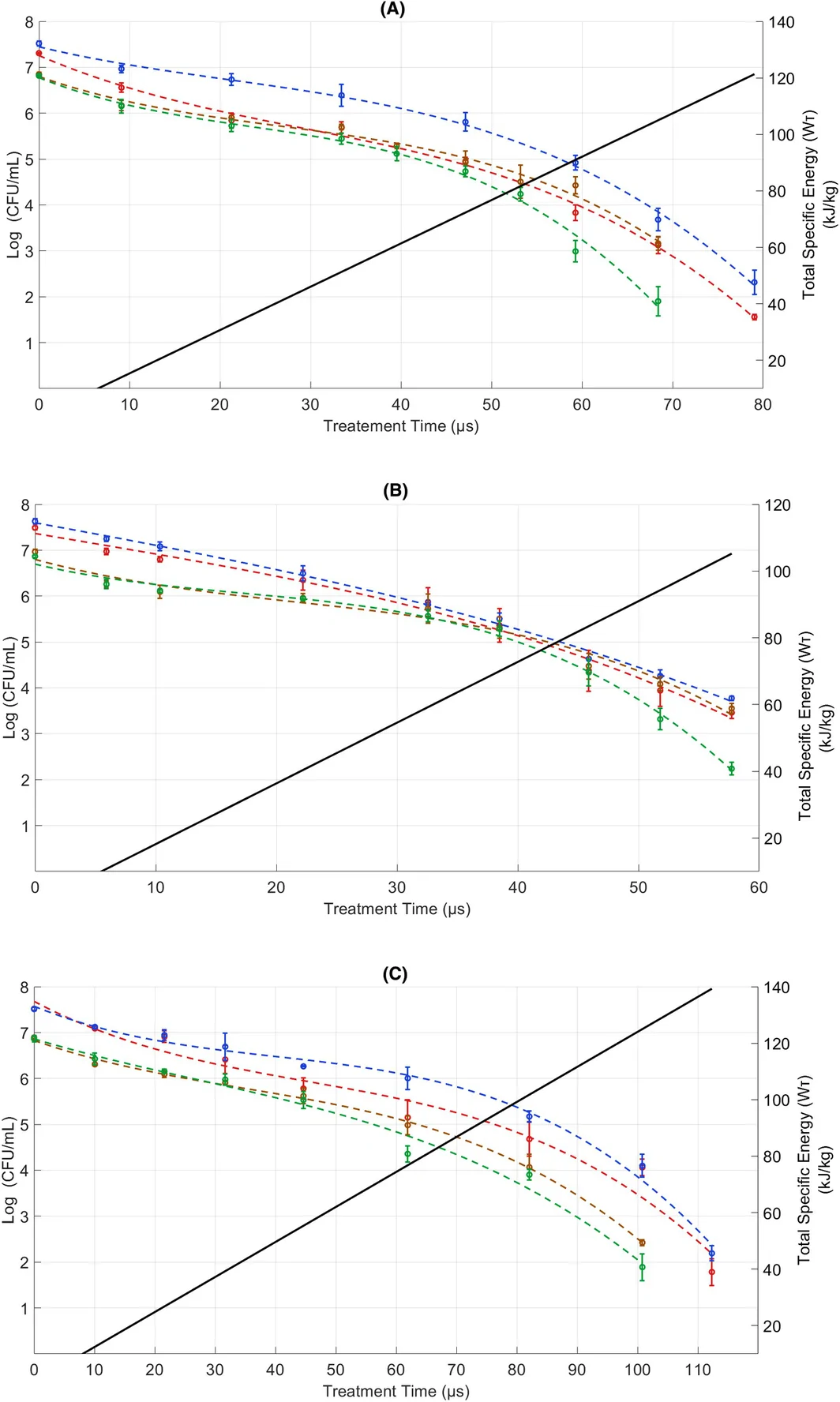
Fitted survival curves of *E. coli* K12 (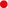), *E. coli* FAM 21843 (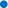), *S. cerevisiae* AD1890 (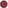) and *S. cerevisiae* 130.0014 (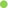) based on experimental data describing the changes in Log CFU/mL with treatment time (μs) and total specific energy (kJ/kg) at pomegranate (A), tomato (B), and carrot (C) juices. Experimental data and error bars represent the mean values of three biological replicates and standard deviations on the log‐transformed data.

In pomegranate juice, the *E. coli* strains followed a concave downward inactivation trend, with a slow initial decline accelerating sharply after 47.12 μs (72.32 kJ/kg). In tomato juice, *E. coli* inactivation approached a near log‐linear pattern with slight tailing at longer treatments, corresponding to modest reductions (< 4.03 and < 3.85 log for K12 and FAM 21843, respectively). Conversely, carrot juice induced a concave upward curve with a pronounced shoulder up to 44.64 μs (55.35 kJ/kg), indicating substantial resistance, followed by rapid microbial decline beyond 61.92 μs (76.78 kJ/kg). These differences among food matrices indicate that potentially the composition of the surrounding environment influenced microbial stress response, with carrot juice showing a greater protective effect during the initial phase of PEF treatment compared with pomegranate and tomato juices.

Regarding *S. cerevisiae* , similar trends were observed. In pomegranate juice, the microbial inactivation of the *S. cerevisiae* followed by a concave downward behavior with a gradual decline that accelerates at higher treatment times/total specific energies. In tomato juice, *S. cerevisiae* strains showed a concave downward behavior observed with a sharper slope, indicating higher sensitivity at PEF treatments of 38.48 μs (70.0 kJ/kg). In carrot juice, the microbial data of *S. cerevisiae* showed an extended “shoulder effect,” indicating lower inactivation at low‐energy PEF treatments followed by higher inactivation at PEF treatments higher than 61.92 μs (76.78 kJ/kg).

The estimated model parameters (*C*_1_, *C*_2_, *C*_3_), along with the regression coefficient (*R* ^2^), Akaike information criterion (*AIC*), and mean square error (*MSE*), are summarized in Table 3. The regression coefficients *R* ^2^ ranged from 0.965 to 0.998 across all datasets, demonstrating a strong model fit, as in general values close to 1 indicate the high accuracy of the model. The *AIC* values varied between −8.17 and 18.21, while the *MSE* values ranged from 0.008 to 0.182 for all strains.

**TABLE 3:** Parameter estimation of the model Equation (5) in pomegranate, tomato, and carrot juices.

Strain	*C*_1_	*C*_2_	*C*_3_	Log *N*_0_ (Log CFU/mL)	*R* ^2^	AIC	MSE	SSE
Pomegranate juice								
*E. coli* K12	(1.96 ± 0.41) × 10^−1^	(−4.7 ± 1.7) × 10^−3^	(5.0 ± 1.4) × 10^−5^	7.26 ± 0.14	0.996	1.42	0.026	0.103
*E. coli* FAM 21843	(1.09 ± 0.33) × 10^−1^	(−2.8 ± 1.3) × 10^−3^	(4.3 ± 1.1) × 10^−5^	7.45 ± 0.11	0.997	−2.38	0.016	0.065
*S. cerevisiae* AD1890	(1.60 ± 0.50) × 10^−1^	(−4.8 ± 2.3) × 10^−3^	(5.8 ± 2.1) × 10^−5^	6.80 ± 0.16	0.986	1.24	0.028	0.138
*S. cerevisiae* 130.0014	(1.81 ± 0.57) × 10^−1^	(−6.2 ± 2.6) × 10^−3^	(8.6 ± 2.4) × 10^−5^	6.77 ± 0.18	0.991	3.67	0.036	0.181
Tomato juice								
*E. coli* K12	(1.12 ± 0.69) × 10^−1^	(−1.0 ± 3.1) × 10^−3^	(2.6 ± 3.1) × 10^−5^	7.39 ± 0.17	0.989	3.43	0.035	0.176
*E. coli* FAM 21843	(1.14 ± 0.43) × 10^−1^	(−1.0 ± 1.9) × 10^−3^	(2.0 ± 1.9) × 10^−5^	7.64 ± 0.10	0.996	−5.13	0.014	0.068
*S. cerevisiae* AD1890	(1.64 ± 0.81) × 10^−1^	(−5.0 ± 3.4) × 10^−3^	(6.3 ± 3.7) × 10^−5^	6.80 ± 0.20	0.976	6.26	0.048	0.241
*S. cerevisiae* 130.0014	(1.44 ± 0.53) × 10^−1^	(−5.6 ± 2.2) × 10^−3^	(9.0.0 ± 0.22) × 10^−5^	6.77 ± 0.13	0.994	−1.38	0.021	0.103
Carrot juice								
*E. coli* K12	(1.60 ± 0.74) × 10^−1^	(−3.7 ± 2.6) × 10^−3^	(3.3 ± 1.9) × 10^−5^	7.68 ± 0.37	0.965	18.21	0.182	0.911
*E. coli* FAM 21843	(1.12 ± 0.35) × 10^−1^	(−3.6 ± 1.2) × 10^−3^	(3.6 ± 0.9) × 10^−5^	7.57 ± 0.18	0.991	4.67	0.040	0.202
*S. cerevisiae* AD1890	(1.06 ± 0.17) × 10^−1^	(−2.6 ± 0.7) × 10^−3^	(3.0 ± 0.5) × 10^−5^	6.83 ± 0.08	0.998	−8.17	0.008	0.031
*S. cerevisiae* 130.0014	(0.91 ± 0.53) × 10^−1^	(−1.4 ± 2.1) × 10^−3^	(2.0 ± 1.7) × 10^−5^	6.86 ± 0.24	0.985	9.83	0.074	0.294

*Note:* Values are expressed as mean ± standard error (SSE). The letters a, b, and c denote groups of values that were compared using statistical analysis.

The estimated model parameter differences of *C*_1_, *C*_2_, and *C*_3_ are presented in Table 4. In general, significant differences were identified only between model parameters *C*_1_ and *C*_3_, while no differences were identified between *C*_2_. The linear coefficient *C*_2_ showed stability across all microbial strains and food matrices, suggesting a conserved linear response mechanism to energy input. This indicates that while the initial susceptibility (*C*_1_) and nonlinear adaptations (*C*_3_) vary, likely due to strain‐food matrix effects, the linear sensitivity of the microbial inactivation in relation to the increasing total specific energy remains consistent.

**TABLE 4:** Statistical differences of model parameters *C*_1_, *C*_2_, *C*_3_ of *E. coli* K12, *E. coli* FAM 21843, *S. cerevisiae* AD1890, and *S. cerevisiae* 130.0014 in pomegranate, tomato, and carrot juices.

Strain	*C*_1_	*C*_2_	*C*_3_
Pomegranate juice			
*E. coli* K12	A	A	AC
*E. coli* FAM 21843	CD	A	AC
*S. cerevisiae* AD1890	B	A	AC
*S. cerevisiae* 130.0014	AB	A	AB
Tomato juice			
*E. coli* K12	CD	A	C
*E. coli* FAM 21843	C	A	C
*S. cerevisiae* AD1890	B	A	AC
*S. cerevisiae* 130.0014	AB	A	A
Carrot juice			
*E. coli* K12	B	A	AC
*E. coli* FAM 21843	C	A	AC
*S. cerevisiae* AD1890	CD	A	BC
*S. cerevisiae* 130.0014	D	A	C

*Note:* The letters A, B, and C denote groups of values that were compared using statistical analysis.

More specifically, the baseline specific inactivation rate, represented by the coefficient *C*_1_, showed statistically significant (*p* < 0.05) variation across the tested strains and food matrices. The microbial strains were grouped into four statistical categories (A–D) based on analysis of variance and post hoc comparisons. *E. coli* K12 in pomegranate juice was classified within group A, indicating a relatively high baseline inactivation rate and thus greater initial susceptibility to treatment. On the other hand, *S. cerevisiae* 130.0014 in carrot juice belonged to group D, representing the lowest observed baseline rate. The pomegranate and tomato juice were under the A and B groups, indicating a greater susceptibility. Interestingly, *E. coli* FAM 21843 showed no differences in the *C*_1_ baseline inactivation, indicating that the microorganism's initial microbial inactivation under PEF is not affected by the food matrix. Differences (*p* < 0.05) were also observed in the quadratic coefficient *C*_3_, which reflects the nonlinear dynamics of microbial inactivation. Strain‐food matrix combinations were clustered into three main groups (A, B and C). More specifically, in tomato juice, differences were identified between the *S. cerevisiae* 130.0014 and the *E. coli* K12 and FAM 21843 which indicates the different behavior of the microorganisms. In addition, *S. cerevisiae* 130.0014 in both tomato and pomegranate juice showed similar curvature of the microbial inactivation, while it was different in carrot juice which may be related to the effect of pH or the composition of the food matrix. Although statistically significant differences (*p* < 0.05) were observed in the *C*_1_ and *C*_3_ coefficients among the different strains and food matrices, the total specific energy primarily appears to govern the microbial inactivation under the tested pulsed electric field (PEF) conditions. Even though subtle strain‐specific variations were observed, particularly in relation to PEF conditions, these differences were not significant enough to affect the overall kinetic model parameters in this study.

Additionally, at Figure 5, curves presenting the inactivation rates, *k*_*max*_, of *E. coli* K12, *E. coli* FAM 21843, *S. cerevisiae* AD1890 and *S. cerevisiae* 130.0014 in the three different food matrices (pomegranate, tomato, and carrot juices) in relation to the total specific energy are presented. Interestingly, the strains showed various inactivation rates with different *k*_*max*_ values under different PEF treatments (total specific energy). More specifically, at all food matrices the inactivation rate (*k*_*max*_) increased predominantly at high total specific energies (> 80 kJ/kg for all juices and independently of the strain), demonstrating that *k*_*max*_ varies according to the total specific energy. Furthermore, the *k*_*max*_ of *S. cerevisiae* 130.0014 was identified to be higher than that of the other strains at high total specific energies (> 80 kJ/kg) in pomegranate and tomato juice, indicating the high sensitivity of the microorganism as the total specific energy increases. Additionally, small differences were observed between the *E. coli* strains in comparison to the *S. cerevisiae* AD1890 in terms of *k*_*max*_ at high‐energy PEF treatments (> 100 kJ/kg), indicating potential sensitivity of the microorganism at high energy levels of PEF treatments, independent of the food matrix. Thus, these results demonstrate that the total specific energy was the primary factor controlling microbial inactivation, while strain and matrix effects became more evident under specific PEF conditions, with specific differences between the strains becoming more evident at higher total specific energy.

**FIGURE 5:**
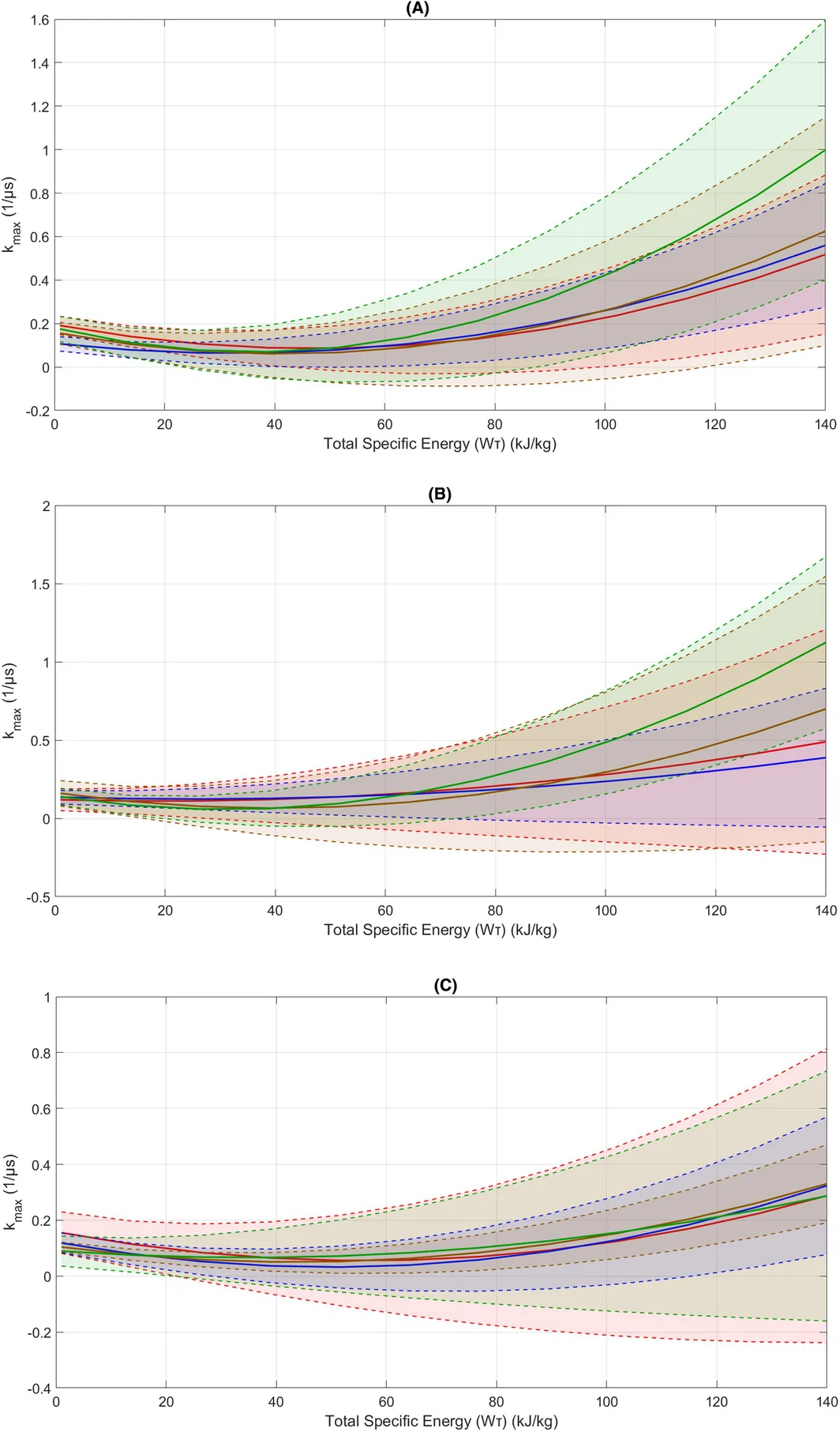
Predicted survival curves of *E. coli* K12 (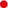), *E. coli* FAM 21843 (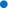), *S. cerevisiae* AD1890 (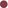) and *S. cerevisiae* 130.0014 (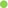) based on model‐derived *k*_*max*_ (1/μs) values as a function of total specific energy (kJ/kg) at pomegranate juice (A), tomato juice (B), and carrot juice (C). The *k*_*max*_ values were theoretically estimated using a fitted second‐order polynomial type of model from experimental data. Dashed lines represent prediction bands obtained via error propagation of the calculated *k*_*max*_ estimates.

### 3.4 Strain Heterogeneity

Overall, K12 and FAM 21843 *E. coli* strains showed similar inactivation behaviors independently of the food matrix. However, in pomegranate juice (pH: 3.1 ± 0.1), minor differences were apparent in the food matrix with the lowest pH, where *E. coli* FAM 21843 showed higher resistance compared to *E. coli* K12. For example, under PEF treatment 47.12 μs, 72.32 *E. coli* K12 and FAM21843 showed log reductions of 2.36 and 1.71, respectively. These findings are consistent with previous works evaluating 10 *E. coli* strains under PEF treatments (20 kV/cm; 88, 136, and 188 kJ/kg) at pH 7.0 (Lytras et al. 2024). In that study, no significant differences (*p* < 0.05) in terms of log reduction were observed between *E. coli* K12 and FAM 21843, and both were identified as among the most resistant strains of the pool. While a study by Gurtler et al. (2010), reported the sensitivity of K12 (ATCC 23716) under PEF treatments (22 kV/cm, 45°C, 59 μs and 20 kV/cm, 55°C, 70 μs) in the acidic environment of orange juice at pH 3.4 (±0.1) in comparison with other strains such as *E. coli* O157:H7 and the surrogate ATCC 35218, indicating a potential sensitivity of the K12 strain under PEF treatments under acidic conditions.

Furthermore, *S. cerevisiae* strains AD1890 and 130.0014 showed differences in their resistance at high total specific energies (> 70–80 kJ/kg), independent of the food matrix. Specifically, *S. cerevisiae* 130.0014 demonstrated higher sensitivity to high‐energy PEF treatments compared to AD1890. For example, the *S. cerevisiae* AD1890 showed a log reduction of 2.42, while the *S. cerevisiae* 130.0014 of 3.82 at PEF treatment (20 kV/cm, 59.28 μs, 90.98 kJ/kg) in pomegranate juice. Similarly, in a previous study where we assessed 10 strains of *S. cerevisiae* under PEF (at 20 kV/cm and 88, 136, and 188 kJ/kg) at neutral pH conditions, we identified that AD1890 and 130.0014 showed statistically significant differences (*p* < 0.05) in terms of log reduction only under the highest PEF treatment condition (20 kV/cm, 188 kJ/kg), indicating potential resistance of the microorganism at high levels of total specific energy compared to other *S. cerevisiae* strains (Lytras et al. 2024). Additionally, a study by Tsagkaropoulou and Karatzas (2024) that investigated the same 10 *S. cerevisiae* strains under 3 conditions of High Pressure Processing (HPP) (200, 300, and 400 MPa) in malt extract broth (MEB) showed that under the lowest HPP parameter (200 MPa), there was no statistical difference (*p* < 0.05) in log reduction between the AD1890 and 130.0014 strains, while under the HPP condition (300 MPa) there was a statistical difference (*p* < 0.05), with AD1890 achieving 2.81 and the 130.0014 5.26 log reductions, mirroring the observed behaviors under PEF treatments and identifying AD1890 as the most resistant strain to HPP. Finally, an investigation assessing a pool of the same 10 *S. cerevisiae* strains under the stress of ultrasound treatment of 255 s revealed that AD1890 was one of the most resistant strains as well (Okafor et al. 2024). This potentially indicates a cross‐resistance of AD1890 to various stresses.

### 3.5 Effect on Quality Characteristics

Regarding the effect on chemical characteristics, analysis of pomegranate, tomato, and carrot juice after respective PEF treatments, were conducted in parallel to microbial inactivation analysis, revealing no significant statistical differences (*p* < 0.05) between the untreated and treated samples in total phenolic content (TPC), total flavonoid content (TFC), total orthodiphenol content (TdOPC), and CUPRAC (antioxidant capacity) values (Supporting Information, Figures S1–S3). The levels of these antioxidant compounds and their associated antioxidant activities remained essentially unchanged following PEF processing, indicating that PEF does not degrade or significantly alter these bioactive compounds in pomegranate, tomato and carrot juice. Similarly, previous studies have reported that PEF treatments have minimal effects to the total phenolic content in pomegranate juice (Guo et al. 2014), tomato juice (Odriozola‐Serrano et al. 2009), carrot juice (Quitão‐Teixeira et al. 2009) and other juices such as apple juice (Noci et al. 2008) and fruit juice mixed beverages (Rodríguez‐Roque et al. 2015). Additionally, Guo et al. (2014) have shown no alteration not only of the total phenolic content, but also of the total anthocyanin content and antioxidant compounds of pomegranate juice in PEF treated samples (35 and 38 kV/cm, 55°C) and in comparison with the untreated sample, while similar behavior to both PEF treated and untreated samples was observed under storage at 4°C for 12 weeks. Furthermore, a study by Odriozola‐Serrano et al. (2008) have shown that there were no statistical differences between PEF‐treated (35 kV/cm, 1500 μs, < 40°C) and untreated samples in terms of the antioxidant through the evaluation of free radical‐scavenging effect on 1,1‐diphenyl‐2‐picrylhydrazyl (DPPH) radical. Overall, this indicates the potential ability of PEF to preserve the nutritional characteristics of the food products under PEF treatments that can lead to efficient microbial inactivation.

## 4 Conclusion

Primarily, this study investigated the microbial inactivation responses of *E. coli* (K12 and FAM 21843) and *S. cerevisiae* (AD1890 and 130.0014) strains to PEF treatments and secondary the PEF effects on the quality characteristics of pomegranate, tomato, and carrot juices. The experimental findings demonstrated that microbial inactivation kinetics under PEF exhibited nonlinear behavior best described by a second‐order polynomial model, with model parameters (*C*_1_, *C*_2_, *C*_3_). The *C*_2_, linear coefficient, did not significantly differ across all the strains, while statistical differences were identified for *C*_1_, baseline inactivation constant, and *C*_3_, quadratic coefficient.

These results indicate that microbial inactivation is primarily determined by the total specific energy, with minor variations influenced by strain‐specific characteristics and the food matrix. Additionally, the microbial inactivation rates (*k*_*max*_) of all the strains showed a gradual increase at higher total specific energies, with *S. cerevisiae* 130.0014 showing higher *k*_*max*_ values compared to the other strains. Minor differences and variations were observed between the strains, particularly at high total specific energies. Regarding the *E. coli* strains, differences were observed under PEF treatments in pomegranate juice, with K12 showing higher sensitivity compared to FAM21843. While both strains showed low sublethal levels under PEF treatments, independent of the juice, with pomegranate juice showing the highest levels. Regarding *S. cerevisiae* strains, AD1890 showed higher resistance at the high total specific energies compared to 130.0014.

Secondly, assessments on the quality characteristics of pomegranate, tomato, and carrot juices revealed no effect on the antioxidant compounds and their associated antioxidant activities. Overall, this study demonstrates that, under the applied PEF conditions, the technology achieved microbial reductions relevant for food preservation while preserving the antioxidant compounds and antioxidant capacity of the juices. In addition, these findings showed that the total specific energy is a key determinant of PEF induced microbial inactivation, while some strain‐ and matrix‐dependent variations have also an important role in determining the treatment efficiency. Future research emphasis should be placed on creating predictive models that account for variations in microbial strains and food matrices. Optimizing PEF conditions to achieve effective microbial control while preserving product quality and evaluating its long‐term effects on juice characteristics are crucial. In addition, other quality attributes such as the color, volatile compounds, flavor, and overall sensory perception should be investigated in future studies to provide a more comprehensive assessment. Addressing these aspects will facilitate the development of more efficient, reliable, and scalable PEF applications in the food industry. Such advancements promote the adoption of PEF technology as an innovative approach that meets consumer demand for fresh‐like, minimally processed juices while enhancing food safety; however, the initial cost of PEF equipment remains a significant criterium for its widespread industrial implementation.

## Author Contributions

**Fotios Lytras:** conceptualization, investigation, methodology, writing – review and editing, writing – original draft, visualization, data curation. **Georgios Psakis:** writing – review and editing, supervision. **Matthew Xuereb:** methodology, writing – review and editing, data curation. **Frederick Lia:** methodology, resources. **Ruben Gatt:** writing – review and editing, supervision, resources. **Javier Raso:** writing – review and editing. **Vasilis Valdramidis:** conceptualization, methodology, writing – review and editing, supervision, resources, funding acquisition, project administration.

## Funding

This project has received funding from the European Union's Horizon 2020 research and innovation programme under the Marie Skłodowska‐Curie grant agreement No 955431.

## Conflicts of Interest

The authors declare no conflicts of interest.

## Supporting information


**Figure S1:** Total phenolic content (TPC) (pink), total flavonoid content (TFC) (yellow), total orthodiphenol content (TdOPC) (blue), and CUPRAC activity (green) under 4 different conditions, including PEF treatments at constant electric field strength 20 kV/cm and total specific energies of 32.66, 72.32, 90.98, and 121.31 kJ/kg in pomegranate juice. Experiments were performed in 3 independently processed samples. Bars represent the standard deviations of these measurements.
**Figure S2:** Total phenolic content (TPC) (pink), total flavonoid content (TFC) (yellow), total orthodiphenol content (TdOPC) (blue), and CUPRAC activity (green) under 4 different conditions, including PEF treatments at constant electric field strength 20 kV/cm and total specific energies of 40.39, 59.24, 83.48, and 105.22 kJ/kg in tomato juice. Experiments were performed in 3 independently processed samples. Bars represent the standard deviations of these measurements.
**Figure S3:** Total phenolic content (TPC) (pink), total flavonoid content (TFC) (yellow), total orthodiphenol content (TdOPC) (blue), and CUPRAC activity (green) under 4 different conditions, including PEF treatments at constant electric field strength 20 kV/cm and total specific energies of 55.35, 101.78, 124.99, and 139.28 kJ/kg in carrot juice. Experiments were performed in 3 independently processed samples. Bars represent the standard deviations of these measurements.
**Figure S4:** Calibration Curve for the determination of Total Phenolic Content (TPC).
**Figure S5:** Calibration Curve for the determination of Total Flavonoid Content (TFC).
**Figure S6:** Calibration Curve for the determination of total orthodiphenol content (TDOPC).
**Figure S7:** Calibration Curve for the determination of CUPRAC Antioxidant activity.

## Data Availability

The data underlying this article are available in the article.

## References

[fsn372408-bib-0001] Alkhafaji, S. , and M. Farid . 2008. “Modelling the Inactivation of *Escherichia coli* ATCC 25922 Using Pulsed Electric Field.” Innovative Food Science and Emerging Technologies 9: 448–454. 10.1016/j.ifset.2008.02.003.

[fsn372408-bib-0002] Álvarez, I. , R. Pagán , J. Raso , and S. Condón . 2002. “Environmental Factors Influencing the Inactivation of *Listeria monocytogenes* by Pulsed Electric Fields.” Letters in Applied Microbiology 35: 489–493. 10.1046/j.1472-765X.2002.01221.x. 12460430

[fsn372408-bib-0003] Álvarez, I. , R. Virto , J. Raso , and S. Condón . 2003. “Comparing Predicting Models for the *Escherichia coli* Inactivation by Pulsed Electric Fields.” Innovative Food Science and Emerging Technologies 4: 195–202. 10.1016/S1466-8564(03)00004-3.

[fsn372408-bib-0004] Amiali, M. , M. O. Ngadi , J. P. Smith , and G. S. V. Raghavan . 2007. “Synergistic Effect of Temperature and Pulsed Electric Field on Inactivation of *Escherichia coli* O157:H7 and *Salmonella enteritidis* in Liquid Egg Yolk.” Journal of Food Engineering 79: 689–694. 10.1016/j.jfoodeng.2006.02.029.

[fsn372408-bib-0005] Battaggia, D. , Y. Yao , M. N. Nierop Groot , T. Abee , and H. M. W. den Besten . 2024. “Impact of Species and Strain Variability on Non‐Thermal Plasma Decontamination Efficacy.” Innovative Food Science and Emerging Technologies 95: 103674. 10.1016/j.ifset.2024.103674.

[fsn372408-bib-0006] Brito, I. P. C. , and E. K. Silva . 2024. “Pulsed Electric Field Technology in Vegetable and Fruit Juice Processing: A Review.” Food Research International 184: 114207. 10.1016/j.foodres.2024.114207. 38609209

[fsn372408-bib-0007] Buzrul, S. , and H. Alpas . 2004. “Modeling the Synergistic Effect of High Pressure and Heat on Inactivation Kinetics of *Listeria innocua* : A Preliminary Study.” FEMS Microbiology Letters 238: 29–36. 10.1016/j.femsle.2004.07.011. 15336399

[fsn372408-bib-0008] Delso, C. , J. M. Martínez , G. Cebrián , S. Condón , J. Raso , and I. Álvarez . 2022. “Microbial Inactivation by Pulsed Electric Fields, Food Engineering Series.” In Pulsed Electric Fields Technology for the Food Industry. Food Engineering Series, edited by J. Raso , V. Heinz , I. Alvarez , et al. Springer. 10.1007/978-3-030-70586-2_5.

[fsn372408-bib-0009] Den Besten, H. M. W. , M. Mataragas , R. Moezelaar , T. Abee , and M. H. Zwietering . 2006. “Quantification of the Effects of Salt Stress and Physiological State on Thermotolerance of *Bacillus cereus* ATCC 10987 and ATCC 14579.” Applied and Environmental Microbiology 72: 5884–5894. 10.1128/AEM.00780-06. PMC1563614 16957208

[fsn372408-bib-0010] Evrendilek, G. A. 2017. “Impacts of Pulsed Electric Field and Heat Treatment on Quality and Sensory Properties and Microbial Inactivation of Pomegranate Juice.” Food Science and Technology International 23: 668–680. 10.1177/1082013217715369. 28610446

[fsn372408-bib-0011] Garcia, D. , P. Manas , N. Gomez , J. Raso , and R. Pagan . 2006. “Biosynthetic Requirements for the Repair of Sublethal Membrane Damage in *Escherichia coli* Cells After Pulsed Electric Fields.” Journal of Applied Microbiology 100: 428–435. 10.1111/j.1365-2672.2005.02795.x. 16478482

[fsn372408-bib-0012] García, D. , N. Gómez , J. Raso , and R. Pagán . 2005. “Bacterial Resistance After Pulsed Electric Fields Depending on the Treatment Medium pH.” Innovative Food Science and Emerging Technologies 6: 388–395. 10.1016/j.ifset.2005.04.003.

[fsn372408-bib-0013] Geeraerd, A. H. , V. P. Valdramidis , and J. F. Van Impe . 2005. “GInaFiT, a Freeware Tool to Assess Non‐Log‐Linear Microbial Survivor Curves.” International Journal of Food Microbiology 102: 95–105. 10.1016/j.ijfoodmicro.2004.11.038. 15893399

[fsn372408-bib-0014] Guo, M. , T. Z. Jin , D. J. Geveke , X. Fan , J. E. Sites , and L. Wang . 2014. “Evaluation of Microbial Stability, Bioactive Compounds, Physicochemical Properties, and Consumer Acceptance of Pomegranate Juice Processed in a Commercial Scale Pulsed Electric Field System.” Food and Bioprocess Technology 7: 2112–2120. 10.1007/s11947-013-1185-6.

[fsn372408-bib-0015] Gurtler, J. B. , R. B. Rivera , H. Q. Zhang , and D. J. Geveke . 2010. “Selection of Surrogate Bacteria in Place of *E. coli* O157:H7 and *Salmonella typhimurium* for Pulsed Electric Field Treatment of Orange Juice.” International Journal of Food Microbiology 139: 1–8. 10.1016/j.ijfoodmicro.2010.02.023. 20223544

[fsn372408-bib-0016] Heinz, V. , I. Alvarez , A. Angersbach , and D. Knorr . 2001. “Preservation of Liquid Foods by High Intensity Pulsed Electric Fields ‐ Basic Concepts for Process Design.” Trends in Food Science and Technology 12: 103–111. 10.1016/S0924-2244(01)00064-4.

[fsn372408-bib-0017] Heinz, V. , S. T. Phillips , M. Zenker , and D. Knorr . 1999. “Inactivation of *Bacillus subtilis* by High Intensity Pulsed Electric Fields Under Close to Isothermal Conditions.” Food Biotechnology 13: 155–168. 10.1080/08905439909549968.

[fsn372408-bib-0018] Ibrahim, S. A. , T. Bor , D. Song , and M. Tajkarimi . 2011. “Survival and Growth Characteristics of *Escherichia coli* O157:H7 in Pomegranate‐Carrot and Pomegranate‐Apple Blend Juices.” Food and Nutrition Sciences 2: 844–851. 10.4236/fns.2011.28116.

[fsn372408-bib-0019] Jaeger, H. , A. Schulz , N. Karapetkov , and D. Knorr . 2009. “Protective Effect of Milk Constituents and Sublethal Injuries Limiting Process Effectiveness During PEF Inactivation of Lb. Rhamnosus.” International Journal of Food Microbiology 134: 154–161. 10.1016/j.ijfoodmicro.2009.06.007. 19589610

[fsn372408-bib-0020] Johnson Esua, O. , D. W. Sun , C. K. Ajani , J. H. Cheng , and K. M. Keener . 2022. “Modelling of Inactivation Kinetics of Escherichia Coli and *Listeria monocytogenes* on Grass Carp Treated by Combining Ultrasound With Plasma Functionalized Buffer.” Ultrasonics Sonochemistry 88: 106086. 10.1016/j.ultsonch.2022.106086. PMC9287556 35830785

[fsn372408-bib-0021] Kotnik, T. , P. Kramar , G. Pucihar , D. Miklavčič , and M. Tarek . 2012. “Cell Membrane Electroporation ‐ Part 1: The Phenomenon.” IEEE Electrical Insulation Magazine 28: 14–23. 10.1109/MEI.2012.6268438.

[fsn372408-bib-0022] Kwon, R. S. , G. Y. Lee , S. Lee , et al. 2024. “Antimicrobial Properties of Tomato Juice and Peptides Against Typhoidal Salmonella.” Microbiol Spectr 12: e03102‐23. 10.1128/spectrum.03102-23 PMC10913428 38289090

[fsn372408-bib-0023] Lan, T. , J. Wang , S. Bao , et al. 2023. “Effects and Impacts of Technical Processing Units on the Nutrients and Functional Components of Fruit and Vegetable Juice.” Food Research International 168: 112784. 10.1016/j.foodres.2023.112784. 37120231

[fsn372408-bib-0024] Lee, S. , A. Han , S. Jo , et al. 2021. “Microbiological Quality and Safety of Commercial Fresh Fruit and Vegetable Juices in Korea.” Lwt 152: 112432. 10.1016/j.lwt.2021.112432.

[fsn372408-bib-0025] Lytras, F. , G. Psakis , R. Gatt , J. Hummerjohann , J. Raso , and V. Valdramidis . 2024. “Evaluation of Strain Variability of Food Microorganisms in Response to Decontamination by Pulsed Electric Fields and Thermal Treatments.” Innovative Food Science and Emerging Technologies 95: 103731. 10.1016/j.ifset.2024.103731.

[fsn372408-bib-0026] Lytras, F. , G. Psakis , R. Gatt , J. Raso , and V. Valdramidis . 2026. “Modelling Gene‐Dependent PEF Resistance of *E. coli* K‐12.” Food Microbiology 137: 105042. 10.1016/j.fm.2026.105042. 41722985

[fsn372408-bib-0027] Mahnič‐Kalamiza, S. , and D. Miklavčič . 2022. “The Phenomenon of Electroporation, Food Engineering Series.” In Pulsed Electric Fields Technology for the Food Industry. Food Engineering Series, edited by J. Raso , V. Heinz , I. Alvarez , et al. Springer. 10.1007/978-3-030-70586-2_3.

[fsn372408-bib-0028] Martins, I. B. A. , A. Rosenthal , G. Ares , and R. Deliza . 2020. “How Do Processing Technology and Formulation Influence Consumers' Choice of Fruit Juice?” International Journal of Food Science and Technology 55: 2660–2668. 10.1111/ijfs.14519.

[fsn372408-bib-0030] Mosqueda‐Melgar, J. , R. M. Raybaudi‐Massilia , and O. Martín‐Belloso . 2007. “Influence of Treatment Time and Pulse Frequency on *Salmonella enteritidis* , *Escherichia coli* and *Listeria monocytogenes* Populations Inoculated in Melon and Watermelon Juices Treated by Pulsed Electric Fields.” International Journal of Food Microbiology 117: 192–200. 10.1016/j.ijfoodmicro.2007.04.009. 17512997

[fsn372408-bib-0031] Niu, H. , M. Zhang , Q. Yu , and Y. Liu . 2024. “Status and Trends of Artificial Intelligence in the R & D of Future Fruit & Vegetable Juice.” Innovative Food Science and Emerging Technologies 97: 103796. 10.1016/j.ifset.2024.103796.

[fsn372408-bib-0032] Noci, F. , J. Riener , M. Walkling‐Ribeiro , D. A. Cronin , D. J. Morgan , and J. G. Lyng . 2008. “Ultraviolet Irradiation and Pulsed Electric Fields (PEF) in a Hurdle Strategy for the Preservation of Fresh Apple Juice.” Journal of Food Engineering 85: 141–146. 10.1016/j.jfoodeng.2007.07.011.

[fsn372408-bib-0033] Odriozola‐Serrano, I. , R. Soliva‐Fortuny , T. Hernández‐Jover , and O. Martín‐Belloso . 2009. “Carotenoid and Phenolic Profile of Tomato Juices Processed by High Intensity Pulsed Electric Fields Compared With Conventional Thermal Treatments.” Food Chemistry 112: 258–266. 10.1016/j.foodchem.2008.05.087.

[fsn372408-bib-0034] Odriozola‐Serrano, I. , R. Soliva‐Fortuny , and O. Martín‐Belloso . 2008. “Changes of Health‐Related Compounds Throughout Cold Storage of Tomato Juice Stabilized by Thermal or High Intensity Pulsed Electric Field Treatments.” Innovative Food Science and Emerging Technologies 9: 272–279. 10.1016/j.ifset.2007.07.009.

[fsn372408-bib-0035] Okafor, E. T. , F. Pavli , J. Hummerjohann , and V. Valdramidis . 2024. “Determination of Strain Variability and Kinetics of Food‐Associated Microorganisms Following Ultrasound Treatment.” Food Research International 196: 114979. 10.1016/j.foodres.2024.114979. 39614466

[fsn372408-bib-0036] Quitão‐Teixeira, L. J. , I. Odriozola‐Serrano , R. Soliva‐Fortuny , A. Mota‐Ramos , and O. Martín‐Belloso . 2009. “Comparative Study on Antioxidant Properties of Carrot Juice Stabilised by High‐Intensity Pulsed Electric Fields or Heat Treatments.” Journal of the Science of Food and Agriculture 89: 2636–2642. 10.1002/jsfa.3767.

[fsn372408-bib-0037] Raso, J. , I. Alvarez , S. Condón , and F. J. Sala Trepat . 2000. “Predicting Inactivation of Salmonella Senftenberg by Pulsed Electric Fields.” Innovative Food Science and Emerging Technologies 1: 21–29. 10.1016/S1466-8564(99)00005-3.

[fsn372408-bib-0038] Raso, J. , W. Frey , G. Ferrari , et al. 2016. “Recommendations Guidelines on the Key Information To Be Reported in Studies of Application of PEF Technology in Food and Biotechnological Processes.” Innovative Food Science and Emerging Technologies 37: 312–321. 10.1016/j.ifset.2016.08.003.

[fsn372408-bib-0039] Reina, L. D. , Z. T. Jin , Q. H. Zhang , and A. E. Yousef . 1998. “Inactivation of *Listeria monocytogenes* in Milk by Pulsed Electric Field.” Journal of Food Protection 61: 1203–1206. 10.4315/0362-028X-61.9.1203. 9766078

[fsn372408-bib-0040] Rivas, A. , F. Sampedro , D. Rodrigo , A. Martínez , and M. Rodrigo . 2006. “Nature of the Inactivation of *Escherichia coli* Suspended in an Orange Juice and Milk Beverage.” European Food Research and Technology 223: 541–545. 10.1007/s00217-005-0234-4.

[fsn372408-bib-0041] Rodrigo, D. , G. V. Barbosa‐Cánovas , A. Martínez , and M. Rodrigo . 2003. “Weibull Distribution Function Based on an Empirical Mathematical Model for Inactivation of *Escherichia coli* by Pulsed Electric Fields.” Journal of Food Protection 66: 1007–1012. 10.4315/0362-028X-66.6.1007. 12801001

[fsn372408-bib-0042] Rodrigo, M. , D. Rodrigo , and P. Ruı . 2003. “Kinetic Model for the Inactivation of *Lactobacillus plantarum* by Pulsed Electric Fields.” International Journal of Food Microbiology 81: 223–229. 10.1016/s0168-1605(02)00247-7 12485748

[fsn372408-bib-0043] Rodríguez‐Roque, M. J. , B. de Ancos , C. Sánchez‐Moreno , M. P. Cano , P. Elez‐Martínez , and O. Martín‐Belloso . 2015. “Impact of Food Matrix and Processing on the In Vitro Bioaccessibility of Vitamin C, Phenolic Compounds, and Hydrophilic Antioxidant Activity From Fruit Juice‐Based Beverages.” Journal of Functional Foods 14: 33–43. 10.1016/j.jff.2015.01.020.

[fsn372408-bib-0044] Roobab, U. , R. M. Aadil , G. M. Madni , and A. E. D. Bekhit . 2018. “The Impact of Nonthermal Technologies on the Microbiological Quality of Juices: A Review.” Comprehensive Reviews in Food Science and Food Safety 17: 437–457. 10.1111/1541-4337.12336. 33350080

[fsn372408-bib-0045] Saldaña, G. , E. Puértolas , S. Condón , I. Álvarez , and J. Raso . 2010a. “Modeling Inactivation Kinetics and Occurrence of Sublethal Injury of a Pulsed Electric Field‐Resistant Strain of *Escherichia coli* and *Salmonella typhimurium* in Media of Different pH.” Innovative Food Science and Emerging Technologies 11: 290–298. 10.1016/j.ifset.2010.01.003.

[fsn372408-bib-0046] Saldaña, G. , E. Puértolas , S. Condón , I. Álvarez , and J. Raso . 2010b. “Inactivation Kinetics of Pulsed Electric Field‐Resistant Strains of Listeria Monocytogenes and *Staphylococcus aureus* in Media of Different pH.” Food Microbiology 27: 550–558. 10.1016/j.fm.2010.01.002. 20417406

[fsn372408-bib-0047] Saldaña, G. , E. Puértolas , N. López , D. García , I. Álvarez , and J. Raso . 2009. “Comparing the PEF Resistance and Occurrence of Sublethal Injury on Different Strains of *Escherichia coli* , * Salmonella typhimurium*, *Listeria monocytogenes* and *Staphylococcus aureus* in Media of pH 4 and 7.” Innovative Food Science and Emerging Technologies 10: 160–165. 10.1016/j.ifset.2008.11.003.

[fsn372408-bib-0048] Saldaña, G. , E. Puértolas , S. Monfort , J. Raso , and I. Álvarez . 2011. “Defining Treatment Conditions for Pulsed Electric Field Pasteurization of Apple Juice.” International Journal of Food Microbiology 151: 29–35. 10.1016/j.ijfoodmicro.2011.07.033. 21880388

[fsn372408-bib-0049] Singh, J. , M. Singh , B. Singh , M. Nayak , and C. Ghanshyam . 2017. “Comparative Analyses of Prediction Models for Inactivation of *Escherichia coli* in Carrot Juice by Means of Pulsed Electric Fields.” Journal of Food Science and Technology 54: 1538–1544. 10.1007/s13197-017-2585-9. PMC5430186 28559613

[fsn372408-bib-0050] Thamsuaidee, A. , E. Schaefer , D. Schneider , C. Siemer , and V. P. Valdramidis . 2024. “Disentangling the Effects of Electroporation and Heat During Pulsed Electric Field (PEF) Processing of Oat‐Based Milk Alternative: A Case Study on Lactiplantibacillus Plantarum Inactivation.” Innovative Food Science and Emerging Technologies 94: 103691. 10.1016/j.ifset.2024.103691.

[fsn372408-bib-0051] Timmermans, R. A. H. , M. N. Nierop Groot , A. L. Nederhoff , M. A. J. S. van Boekel , A. M. Matser , and H. C. Mastwijk . 2014. “Pulsed Electric Field Processing of Different Fruit Juices: Impact of pH and Temperature on Inactivation of Spoilage and Pathogenic Micro‐Organisms.” International Journal of Food Microbiology 173: 105–111. 10.1016/j.ijfoodmicro.2013.12.022. 24418831

[fsn372408-bib-0053] Toepfl, S. 2012. “Pulsed Electric Field Food Processing‐Industrial Equipment Design and Commercial Applications.” Stewart Postharvest Review 8: 1–7. 10.2212/spr.2012.2.4.

[fsn372408-bib-0054] Tsagkaropoulou, T. , and K. A. G. Karatzas . 2024. “Microbial Species and Strain Heterogeneity Affect Resistance to High Pressure Processing.” Innovative Food Science and Emerging Technologies 94: 103645. 10.1016/j.ifset.2024.103645.

[fsn372408-bib-0055] U.S. Food and Drug Administation . 2000. “No Title [WWW Document]. CFR ‐ Code of Federal Regulations Title 21.” URL. https://www.accessdata.fda.gov/scripts/cdrh/cfdocs/cfcfr/cfrsearch.cfm?fr=179.26.

[fsn372408-bib-0056] Verhaegen, B. , K. De Reu , M. Heyndrickx , I. Van Damme , and L. De Zutter . 2015. “Growth of Stressed Strains of Four Non‐O157 Shiga Toxin‐Producing *Escherichia coli* Serogroups in Five Enrichment Broths.” Journal of Food Protection 78: 1960–1966. 10.4315/0362-028X.JFP-15-019. 26555518

[fsn372408-bib-0058] Wang, F. , L. Li , X. Li , X. Hu , and B. Zhang . 2022. “Pulsed Electric Field Promotes the Growth Metabolism of Aerobic Denitrifying Bacteria *Pseudomonas putida* W207‐14 by Improving Cell Membrane Permeability.” Environmental Technology 44, no. 15: 1–38. 10.1080/09593330.2022.2027028. 35001840

[fsn372408-bib-0059] Westfall, P. H. , R. D. Tobias , and D. Rom . 2000. Multiple Comparisons and Multiple Tests (Text and Workbook Set). Sas Inst.

[fsn372408-bib-0060] Wouters, P. C. , I. Alvarez , and J. Raso . 2001. “Critical Factors Determining Inactivation Kinetics by Pulsed Electric Field Food Processing.” Trends in Food Science and Technology 12: 112–121. 10.1016/S0924-2244(01)00067-X.

[fsn372408-bib-0061] Wright, S. P. 1992. “Adjusted P‐Values for Simultaneous Inference.” Science 48: 1005–1013. 10.1126/science.87.2249.103-a.

[fsn372408-bib-0062] Xuereb, M. A. , G. Psakis , K. Attard , F. Lia , and R. Gatt . 2025. “A Comprehensive Analysis of Non‐Thermal Ultrasonic‐Assisted Extraction of Bioactive Compounds From Citrus Peel Waste Through a One‐Factor‐At‐a‐Time Approach.” Molecules 30: 6–10. 10.3390/molecules30030648. PMC11820553 39942752

[fsn372408-bib-0063] Zhao, W. , R. Yang , H. Q. Zhang , W. Zhang , X. Hua , and Y. Tang . 2011. “Quantitative and Real Time Detection of Pulsed Electric Field Induced Damage on *Escherichia coli* Cells and Sublethally Injured Microbial Cells Using Flow Cytometry in Combination With Fluorescent Techniques.” Food Control 22: 566–573. 10.1016/j.foodcont.2010.10.006.

[fsn372408-bib-0065] Zhong, K. , F. Chen , Z. Wang , J. Wu , X. Liao , and X. Hu . 2005. “Inactivation and Kinetic Model for the *Escherichia coli* Treated by a Co‐Axial Pulsed Electric Field.” European Food Research and Technology 221: 752–758. 10.1007/s00217-005-0015-0.

