# Supplementary material for: Kinetic Modeling of Microbial Inactivation in Juices Treated With Pulsed Electric Fields (PEF): A Comparative Study on Microbial Heterogeneity and Food Matrix Effects
Source: Food Sci Nutr. 2026 Oct 7;14(10):e72408. doi: 10.1002/fsn3.72408 (PMC13643371; doi:10.1002/fsn3.72408)
Supplement: Supplementary file 1 [file FSN3-14-e72408-s001.docx]

Figure S1. Total phenolic content (TPC) (pink), total flavonoid content (TFC) (yellow), total orthodiphenol content (TdOPC) (blue), and CUPRAC activity (green) under 4 different conditions, including PEF treatments at constant electric field strength 20 kV/cm and total specific energies of 32.66, 72.32, 90.98, and 121.31 kJ/kg in pomegranate juice. Experiments were performed in 3 independently processed samples. Bars represent the standard deviations of these measurements.

Figure S2. Total phenolic content (TPC) (pink), total flavonoid content (TFC) (yellow), total orthodiphenol content (TdOPC) (blue), and CUPRAC activity (green) under 4 different conditions, including PEF treatments at constant electric field strength 20 kV/cm and total specific energies of 40.39, 59.24, 83.48, and 105.22 kJ/kg in tomato juice. Experiments were performed in 3 independently processed samples. Bars represent the standard deviations of these measurements.

Figure S3. Total phenolic content (TPC) (pink), total flavonoid content (TFC) (yellow), total orthodiphenol content (TdOPC) (blue), and CUPRAC activity (green) under 4 different conditions, including PEF treatments at constant electric field strength 20 kV/cm and total specific energies of 55.35, 101.78, 124.99, and 139.28 kJ/kg in carrot juice. Experiments were performed in 3 independently processed samples. Bars represent the standard deviations of these measurements.

Abs

| Conc of Gallic Acid mg/l | TPC |
| --- | --- |
| 0 | 0.05 |
| 0 | 0.053 |
| 0 | 0.047 |
| 0 | 0.047 |
| 0 | 0.05 |
| 0 | 0.048 |
| 5 | 0.068 |
| 5 | 0.072 |
| 5 | 0.07 |
| 5 | 0.075 |
| 5 | 0.084 |
| 5 | 0.071 |
| 10 | 0.086 |
| 10 | 0.112 |
| 10 | 0.088 |
| 10 | 0.1 |
| 10 | 0.087 |
| 10 | 0.087 |
| 25 | 0.122 |
| 25 | 0.114 |
| 25 | 0.116 |
| 25 | 0.133 |
| 25 | 0.121 |
| 25 | 0.126 |
| 50 | 0.192 |
| 50 | 0.209 |
| 50 | 0.198 |
| 50 | 0.211 |
| 50 | 0.231 |
| 50 | 0.211 |

Figure S4. Calibration Curve for the determination of Total Phenolic Content (TPC)

TFC

Absorbance (Abs)

| Quercetin mg/L | TFnC |
| --- | --- |
| 0 | 0.092 |
| 0 | 0.094 |
| 0 | 0.1 |
| 0 | 0.111 |
| 0 | 0.111 |
| 0 | 0.121 |
| 5 | 0.119 |
| 5 | 0.119 |
| 5 | 0.117 |
| 5 | 0.113 |
| 5 | 0.121 |
| 5 | 0.109 |
| 10 | 0.139 |
| 10 | 0.139 |
| 10 | 0.143 |
| 10 | 0.132 |
| 10 | 0.15 |
| 10 | 0.144 |
| 25 | 0.155 |
| 25 | 0.175 |
| 25 | 0.181 |
| 25 | 0.176 |
| 25 | 0.186 |
| 25 | 0.197 |
| 50 | 0.264 |
| 50 | 0.248 |
| 50 | 0.251 |
| 50 | 0.256 |
| 50 | 0.249 |
| 50 | 0.255 |

Figure S5. Calibration Curve for the determination of Total Flavonoid Content (TFC)

Absorbace (abs)

| Protocatechuic acid mg/l | TDOPC |
| --- | --- |
| 0 | 0.054 |
| 0 | 0.051 |
| 0 | 0.052 |
| 0 | 0.052 |
| 0 | 0.057 |
| 0 | 0.054 |
| 5 | 0.059 |
| 5 | 0.062 |
| 5 | 0.062 |
| 5 | 0.06 |
| 5 | 0.063 |
| 5 | 0.061 |
| 10 | 0.065 |
| 10 | 0.074 |
| 10 | 0.066 |
| 10 | 0.072 |
| 10 | 0.064 |
| 10 | 0.067 |
| 25 | 0.086 |
| 25 | 0.075 |
| 25 | 0.085 |
| 25 | 0.084 |
| 25 | 0.08 |
| 25 | 0.083 |
| 50 | 0.118 |
| 50 | 0.108 |
| 50 | 0.114 |
| 50 | 0.12 |
| 50 | 0.124 |
| 50 | 0.122 |

Figure S6. Calibration Curve for the determination of total orthodiphenol content (TDOPC)

CUPRAC

Abs

| Gallic acid mg/L | Cupric |
| --- | --- |
| 0 | 0.168 |
| 0 | 0.165 |
| 0 | 0.184 |
| 0 | 0.13 |
| 0 | 0.147 |
| 0 | 0.166 |
| 5 | 0.207 |
| 5 | 0.217 |
| 5 | 0.195 |
| 5 | 0.206 |
| 5 | 0.211 |
| 5 | 0.223 |
| 10 | 0.258 |
| 10 | 0.238 |
| 10 | 0.25 |
| 10 | 0.256 |
| 10 | 0.269 |
| 10 | 0.258 |
| 25 | 0.306 |
| 25 | 0.325 |
| 25 | 0.319 |
| 25 | 0.333 |
| 25 | 0.351 |
| 25 | 0.286 |
| 50 | 0.453 |
| 50 | 0.443 |
| 50 | 0.464 |
| 50 | 0.459 |
| 50 | 0.474 |
| 50 | 0.459 |

Figure S7. Calibration Curve for the determination of CUPRAC Antioxidant activity
